# New Insights into the Potential of Endogenous Redox Systems in Wheat Bread Dough

**DOI:** 10.3390/antiox7120190

**Published:** 2018-12-12

**Authors:** Nicolas Navrot, Rikke Buhl Holstborg, Per Hägglund, Inge Lise Povlsen, Birte Svensson

**Affiliations:** 1 Enzyme and Protein Chemistry (EPC), Department of Systems Biology, Technical University of Denmark, Building 224, DK-2800 Kgs Lyngby, Denmark; pmh@sund.ku.dk (P.H.); bis@bio.dtu.dk (B.S.); 2 Institute of Plant Molecular Biology, CNRS UPR 2357, University of Strasbourg, 12 rue du Général Zimmer, 67084 Strasbourg CEDEX, France; 3 DuPont Nutrition Biosciences ApS, Edwin Rahrs Vej 38, DK-8220 Brabrand, Denmark; Rikke.Buhl.Holstborg@dupont.com (R.B.H.); inge.lise.povlsen@arlafoods.com (I.L.P.); 4 Department of Biomedical Sciences, University of Copenhagen, Blegdamsvej 3, DK-2200 København N, Denmark; 5 Arla Food Ingredients, Sønderhøj 12, DK-8260 Viby J, Denmark

**Keywords:** wheat, thioredoxin, thioredoxin reductase, baking, redox, dough rheology

## Abstract

Various redox compounds are known to influence the structure of the gluten network in bread dough, and hence its strength. The cereal thioredoxin system (NTS), composed of nicotinamide adenine dinucleotide phosphate (NADPH)-dependent thioredoxin reductase (NTR) and thioredoxin (Trx), is a major reducing enzymatic system that is involved in seed formation and germination. NTS is a particularly interesting tool for food processing due to its heat stability and its broad range of protein substrates. We show here that barley NTS is capable of remodeling the gluten network and weakening bread dough. Furthermore, functional wheat Trx that is present in the dough can be recruited by the addition of recombinant barley NTR, resulting in dough weakening. These results confirm the potential of NTS, especially NTR, as a useful tool in baking for weakening strong doughs, or in flat product baking.

## 1. Introduction

**Figure 1:**
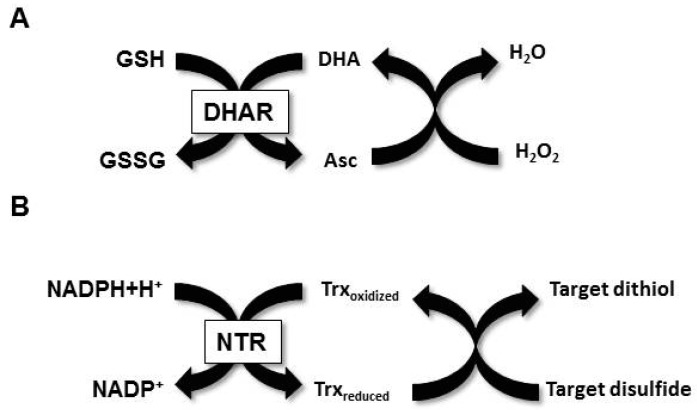
Ascorbate- and thioredoxin-dependent reducing pathways. (**A**) Dehydroascorbate reductase (DHAR) regenerates reduced ascorbate by oxidizing reduced glutathione (GSH) to disulfide-linked oxidized glutathione (GSSG). Ascorbate (Asc) can then be oxidized back to dehydroascorbate (DHA) upon reaction with a molecule H_2_O_2_ or other oxidants. (**B**) Reduced Trx regenerates an oxidized target disulfide bond to a dithiol. Trx itself is then reduced to its active form by a nicotinamide adenine dinucleotide phosphate (NADPH)-dependent thioredoxin reductase.

Bread is a basic element of the daily diet all over the world, and standard production procedures are important for achieving reproducible quality in various production sites. As the raw material shows natural differences in many qualitative aspects, such as protein content, it has been crucial to develop tools to manipulate the bread properties to compensate for natural variations between wheat cultivars, or to adapt flour for specific applications. As an example, the variation in high molecular weight (HMW) glutenin contents in different flours influences the elasticity of the dough [1]. An accurate assessment of the variations found in flours from different wheat cultivars is thus essential in order to understand and predict dough properties [2]. Sulfur-containing molecules such as glutathione and cysteine are known to influence the redox state of the gluten network in the dough, and hence its strength [3]. In this context, small redox active molecules such as ascorbate, glutathione, or cysteine, and enzymes such as oxidases have been investigated in the past. Berland and Launey [4] showed that ascorbate and glutathione (GSH) strengthen and weaken bread dough, respectively. GSH is thought to suppress the disulfide formation of gluten proteins by binding to thiol groups of cysteine residues, mostly in glutenins [5]. Ascorbate acts indirectly by causing GSH depletion through the activity of the endogenous dehydroascorbate reductase enzyme (DHAR, also referred to as glutathione dehydrogenase), which uses GSH to reduce ascorbate that is oxidized by H_2_O_2_ and other reactive oxygen species [6] (Figure 1A). In contrast, the addition of GSH in gluten-free systems, e.g., rice batters, yielded an increased bread volume, due to the reduction of disulfides in the protein network [7]. The addition of cysteine reduces dough strength and augments its fluidity, and cysteine is considered by the FDA to be a GRAS ingredient of up to concentrations of 90 ppm in flour, although its chemical or animal origin is a slight drawback to its use [8]. Plant thiol-based reducing and oxidizing enzymes are thus to be considered when looking for indicators or tools for dough improvement, especially when looking for natural, non-animal origin additives [9].

Nicotinamide adenine dinucleotide phosphate (NADPH)-dependent thioredoxin reductase (NTR) and thioredoxin (Trx) constitute a versatile multi-enzymatic NTS that has been extensively studied in plants. NTS functions by transferring reducing power from NADPH to various target proteins via a Trx-mediated disulfide bridge reduction [10] (Figure 1B). Plant genomes typically contain two NTR-encoding genes and more (up to 20) Trx isoforms, which in all cell types are involved in the regulation of enzymes from the normal metabolism, as well as stress-related responses [11,12]. Proteomics studies identified numerous such potential targets, of which several are only observed at the protein level [13]. Several Trx targets are known in cereal seeds, e.g., barley α-amylase/subtilisin inhibitor (BASI), and proteomics methods have been developed to expand knowledge of these systems [14]. Trx received interest in the field of food chemistry due to its broad range of protein substrates, and to its robustness. NTS, in particular, was investigated with regard to its ability to reduce the allergenicity of bread and other food products [15,16]. Indeed, redox biochemistry in food is far from being well understood, and most likely, these multi-enzyme systems behave differently according to the process studied.

Plant Trx are highly expressed in seeds, and these very stable proteins likely remain functional in raw dough. In contrast, upstream reducing enzymes, e.g., the flavoprotein NTR, are rather unstable enzymes, and are likely to be lost during industrial flour processing. We therefore hypothesize that endogenous Trx that is present in wheat dough could be used as a natural reducing agent to modify dough properties, if supplemented with reducing equivalents provided by NADPH and barley NTR. We examined these hypotheses here in an experimental dough- and bread-making system.

## 2. Materials and Methods

Raw material. Wheat flour (Reform flour, batch No. 1009160013, protein content 10.9%) and the yeast strain (*Saccharomyces cerevisiae*) were provided by Danisco.

Chemicals. NADPH and Ellman’s reagent were purchased from Sigma-Aldrich (St. Louis, MO, USA). Rabbit antibodies raised against wheat Trx h1 were a kind gift from Bob B. Buchanan (UC Berkeley, CA, USA).

Enzymes. Recombinant barley NTR (HvNTR2) and Trx (HvTrxh1) were produced and purified as previously described [17,18]. The specific activity and detailed kinetic properties of these proteins have been described previously [17,18,19], and it has been demonstrated that HvNTR2 and the homologue HvNTR1 show similar catalytic efficiencies with the two Trx h isozymes, HvTrxh1 and HvTrxh2.

Dough preparation. Flour (50 g for rheological experiment, 300 g for baking experiments) was mixed with water to 55% water absorption using method AACC 54-21 and 2% (bakers’ percentage) NaCl on flour weigh. NADPH and either or both NTR and Trx were then added, and the dough was mixed for 6 min. Dough strength was monitored until a stable value of ca. 400 Brabender units (BU) was reached, as measured by farinograph. Yeast (6% Malterserkors yeast on flour) and glucose (2% on flour) were included in the 300 g samples used in the baked products experiments. NADPH, NTR, Trx, and 10 mM Na-phosphate buffer (pH 6) were kept separately on ice, and mixed just before their addition to the flour in the mixing chamber, immediately after water addition.

Kiffer rig measurements. After mixing, the dough was shaped into a bun, of which samples were cut out for Kiffer rig measurements. Eight dough strips were shaped in a specifically designed mold, and left to rest at 25 °C for 30 min in a tightly closed plastic bag (unless stated otherwise). Eight strips per sample were then assayed by measuring Kieffer uniaxial extension using a Stable Micro Systems model TA-XT2i texture analyzer. The resistance of the dough strip (in g) and the maximum elongation (length in mm, just before the strip broke) were recorded using the Texture Expert Exceed version 2.64 software.

Baked products. Minibreads and buns from yeast-containing samples were prepared by mixing 15 and 50 g of initial flour, respectively, to 450 BU. After fitting into molds, minibreads and buns were proofed at 34 °C, 85% relative humidity (RH) for 55 min, to allow for dough development, then minibread dough stability was tested by sliding half of them down a custom-made slide (“shock treatment”), and finally the products were baked at 220 °C for 8 min (MIWE aeromat CS oven). For enzyme testing, to the flour mix (300 g) was added either buffer (10 mM sodium phosphate pH 6) or a mix of 16 mg NADPH and 2 mg NTR in buffer. The weights and volumes of the minibreads and buns were measured after cooling down. The section and bottom surfaces of four minibreads and buns for each treatment were measured from scanned sections, using ImageJ software.

Dough protein extraction and thiol titration. Dough samples were frozen in liquid N_2_ after mixing and development. Samples were freeze-dried overnight and ground to powder using a mortar and pestle. Sixty milligrams of powder was used for protein extraction (0.5 mL of extraction buffer = 30 mM Tris-HCl pH 8, 200 mM NaCl, 10% glycerol, 0.2% Tween 20) was added. Samples were incubated for 2 hr at 4 °C with shaking, centrifuged at 14,000 rpm for 20 min, and supernatants were collected (soluble protein fraction). Twenty milligrams of protein were loaded and run on NuPAGE Novex 12–15% Bis-Tris minigels (Invitrogen, Carlsbad, CA, USA), and stained with Coomassie blue.

Volumes of 20 and 200 µL of extract were used to measure free thiols with Ellman’s reagent. Briefly, the sample and 5 µL 20 mM DTNB in ethanol were mixed in 10 mM Tris pH 8 buffer (total volume 500 µL). After 30 min in the dark, A_412_ was recorded, and the thiol concentration was calculated from ε_M_ = 13,600 M^−1^cm^−1^ for free TNB anion, and the results were normalized by setting a value of 100 for the control sample.

Western blot. A total of 30 mg soluble protein were loaded and run on NuPAGE Novex 12–15% Bis-Tris minigels (Invitrogen), and blotted onto Hybond-ECL membranes (GE Healthcare, Chicago, IL, USA). The quality of the protein transfer was checked by Ponceau staining, and membranes were afterwards incubated overnight in blocking solution consisting of 2% bovine serum albumine (BSA) in 30 mM Tris pH 8, 200 mM NaCl, 0.05% Tween 20 buffer (TBST). Membranes were incubated with 1/2500 diluted anti-wheat Trx rabbit primary antibody in blocking solution for 2 hr, washed three times for 10 min in TBST, and incubated for 1 hr in 1/2500 diluted secondary goat anti-rabbit horseradish peroxidase-coupled antibody (Dako Danmark A/S, Glostrup, Denmark). After three washes with TBST, membranes were put in plastic film, and an ECL Plus kit (GE Healthcare) and a UVP-Bioimaging system (AH Diagnostics, Helsinki, Finland) were used for detection.

## 3. Results and Discussion

### 3.1. Effect of NTS on Dough Rheological Properties

**Figure 2:**
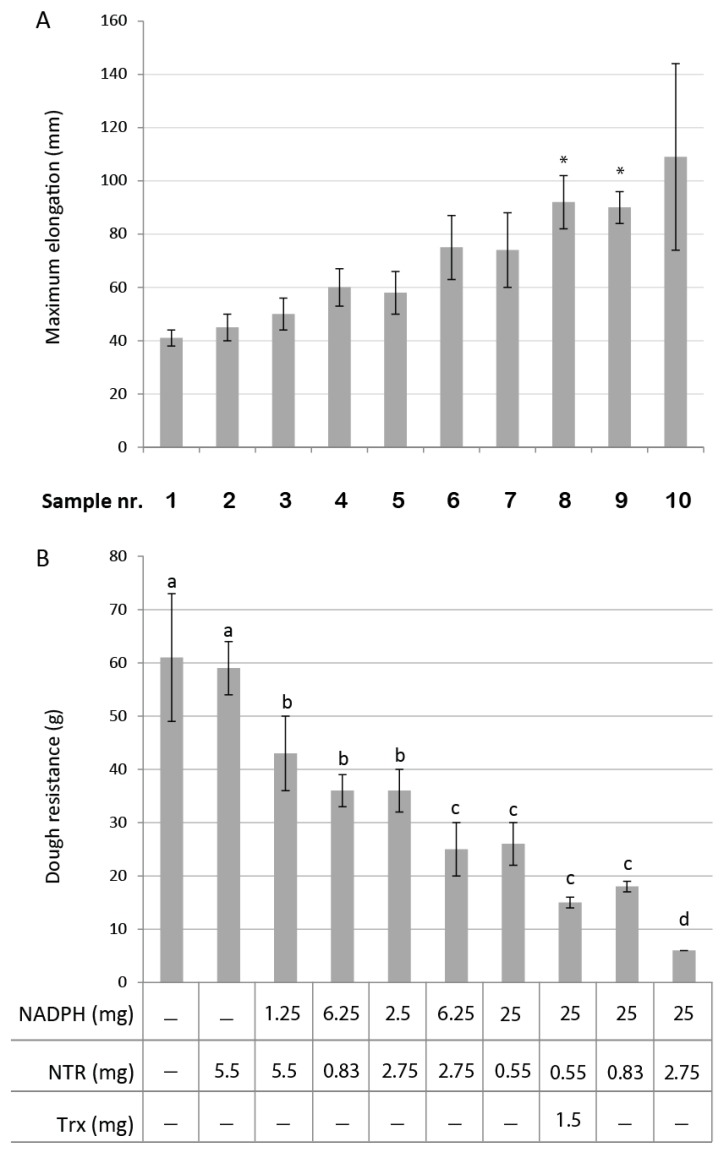
Kieffer rig rheological measurements of dough upon treatment with various amounts of nicotinamide adenine dinucleotide phosphate (NADPH), NADPH-dependent thioredoxin reductase (NTR), thioredoxin (Trx). (**A**) Maximal elongation obtained in mm. Asterisks indicate values that are significantly different from sample 1 (control) (Tukey test, *n* = 6, *p* < 0.05). Sample 10 was excluded from statistical analysis due to high variance on the only two measured values. (**B**) Dough resistance in *g*. Values are the average of 6–8 measurements ± standard deviation, except sample 10, for which *n* = 2. Different letters indicate significant differences (Tukey test, *n* = 6, *p* ≤ 0.05).

As a starting point, a concentration of thiols was chosen based on the 90 ppm cysteine FDA regulation, taking into account that our enzymatic system would probably be less active in the non-optimal dough environment (pH, water content). An amount equivalent to 300 ppm thiol was added in the form of 120 mg Trx, and 13 mg NTR with 140 mg NADPH, for a 50 g flour assay. The amounts of each component were then progressively decreased and mixed in various combinations to assess the effects of the different compounds on the dough system. NADPH and NTR were observed to exert strong effects on the rheological properties of the dough (Figure 2). Control experiments with only buffer or NTR in the absence of NADPH gave reproducible values of ca. 60 g and 40 mm for dough resistance and maximal elongation, respectively (samples 1 and 2). The first assay using amounts corresponding to 300 ppm free thiol (sample 10: 25 mg NADPH plus 2.75 mg NTR) resulted in very soft and sticky dough with barely measurable properties (only two samples in this experiment gave non-aberrant values). Reproducible and measurable values were obtained for the other combinatorial assays, with variable amounts of NADPH and NTR, and as little as 1.25 mg NADPH and 5.5 mg NTR for 50 g flour significantly reduced the resistance and augmented the extensibility of the dough. This effect appeared to be limited by NADPH as well as NTR, as a stronger effect was observed by increasing the amount of either of these two. Additional softening was found when including the remaining NTS component, barley thioredoxin h1 (HvTrxh1), which is known to be regenerated by NTR.

### 3.2. Mini Breads and Buns

**Figure 3:**
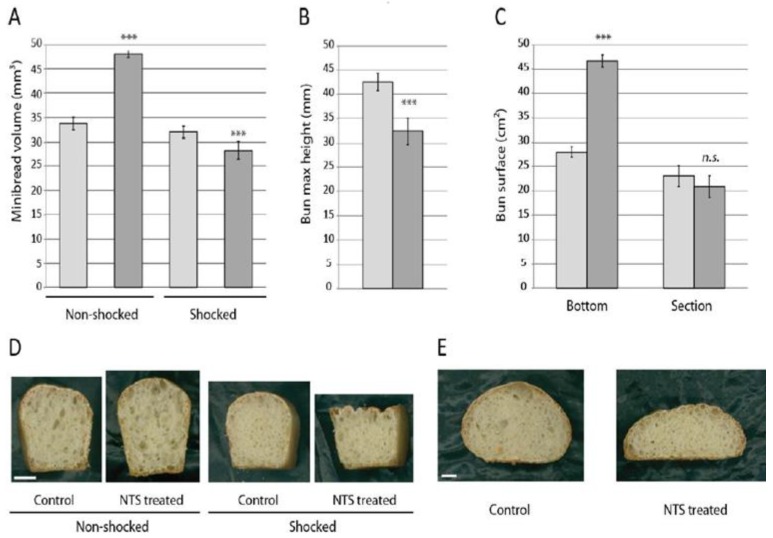
Shape parameters of buns and minibreads treated with NADPH and NTR. The average values for four buns or five minibreads ± standard error are shown. Control: light grey. NTS-treated samples: dark grey. (**A**) Bottom and cross-section surfaces of buns (cm^2^). (**B**) Height of buns (mm). (**C**) Minibread volume (mm^3^). (**D**) Cross-section photography of representative buns treated with phosphate buffer only (control) or 16 mg NADPH and 2 mg NTR in buffer (treated). (**E**) Cross-section photography of representative minibreads treated with phosphate buffer only (control) or 16 mg NADPH and 2 mg NTR in buffer (treated), with or without shock treatment. Asterisks indicate significant difference to control (Student *t*-test, *p* ≤ 0.01). Scale bar = 10 mm.

Further tests were conducted using yeast-containing dough. The influence of NTS was first assessed on minibreads, i.e., 15 g fresh dough baked in tin molds, yielding 11.6 g ± 0.2 g of baked product. Addition of NTR and NADPH to the dough clearly increased the minibread volume, due to increased height (Figure 3A). This indicates that our exogenous NTS was active and effective during dough resting, in addition to yeast metabolism. The increase in volume was explained by the relaxation of the gluten network, allowing for further expansion of CO_2_ bubbles. This effect was suppressed when a light shock treatment was applied, resulting in dramatic collapse of the dough, due to the weakened gluten network. In this case, the measured height dropped below those seen for the control dough. When no enzyme was added to the dough, the shock treatment had no significant effect on minibread volume (Figure 3A,D).

In case of free-standing rolls (50 g fresh weight, 39 g ± 1.1 g baked product) we only analyzed the effect of enzyme addition. This addition caused an increase in the bottom surface of the rolls, while the maximum height decreased (Figure 3B,C). This confirmed our rheological tests on dough, i.e., the dough is softened and more extensible upon the addition of NTS. The loss in height was somehow compensated by an increased bottom surface, resulting in a change in the shape of the roll, which was flatter upon enzyme treatment, and a slight loss of volume (Figure 3E).

### 3.3. Protein Extraction and Dough Thiol Content

**Figure 4:**
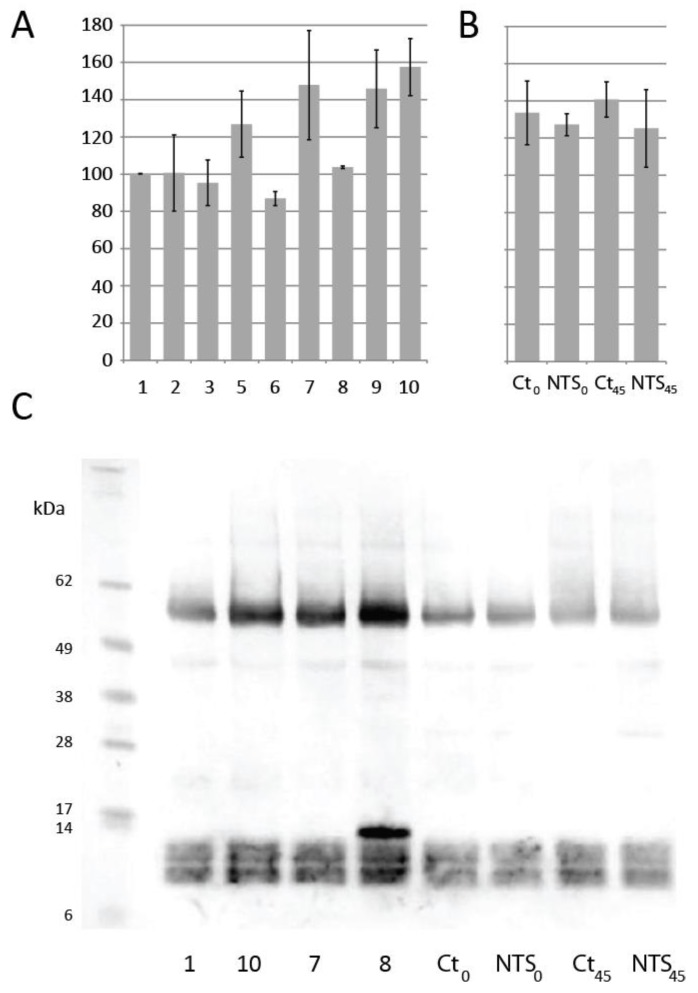
(**A**) Dough soluble thiol content measured as by Ellmann reagent in samples 1 to 10, corresponding to the conditions detailed in Figure 2. (**B**) Yeast-containing dough soluble thiol content measured as by Ellmann reagent in dough used for baking tests. Time point 0 corresponds to dough sampled just after mixing, and *t* = 45 min to dough just before baking, after development. Control sample value was set to 100%. (**C**) Western blot profile of dough extract with anti-wheat Trxh1 polyclonal antibodies. A marker in the first lane (size in kDa) was added for reference from the picture of the Ponceau-stained membrane before immunodetection.

Dough samples were snap-frozen in liquid nitrogen, and ground with a mortar and pestle. After the addition of phosphate buffer, the soluble fraction was analyzed by sodium dodecyl sulfate–polyacrylamide gel electrophoresis (SDS-PAGE) and Western blot, and free thiols were also quantified. Quantification showed a higher content of free thiols that were extractible from the dough when it was treated with high amounts of NTS (Figure 4A). Unfortunately, no statistical significant difference could be calculated, due to the small number (*n* = 2) of measurements done. In yeast-containing dough, the free thiol content was higher than in samples without yeast, and NTS seemed to induce a slight decrease in thiol content (Figure 4B). Western blots using anti-wheat Trxh1 detected recombinant barley Trxh1 in those dough samples to which it was added (Figure 4C). This cross-species reactivity was previously observed [17,20,21,22]. A strong background signal was observed, and it prevented the detection of endogenous wheat Trx that was present in the flour.

## 4. Conclusions

We showed in this work that NTS is a powerful tool for the remodeling of the gluten network and the weakening of the dough, likely through functional wheat Trx being recruited in the dough upon addition of recombinant NTR. The addition of NTS induced dough weakening, as expected from reducing agents. It was possible to extract protein from dough and detect recombinant Trx spiked in the dough, together with a background signal from other proteins including most probably wheat Trx. Soluble dough extracts were also used to estimate the contents of soluble free thiols in the dough, which likely increased when NTS was added. Furthermore, this softening both of dough with and without yeast showed that added NTR and Trx activity were not lost due to elevated yeast metabolism in the developing dough. It is suggested that the optimization of the systems can involve increasing amounts of NTR added to the mix. The synergistic effect of Trx will allow for the reduction of the levels of NADPH needed for efficacy, and lead to a cost-effective procedure to soften strong flour systems using endogenous cereal NTR and Trx, where dough weakening is required, e.g., in flat product baking or laminated dough. Relaxation of the gluten network by enzymatic reduction is also increasing the volume of developed dough, although the shock sensibility observed here is a severe issue for any application in this direction. Using the NTS system, the costly addition of NADPH could be circumvented via the development of yeast strains containing high endogenous NTS system activity and/or to strains designed for specific applications, such as pizza or laminated dough making. The development of yeast GSH biosynthetic pathway mutants has already shown increases in their contents of GSH and derivatives [23], opening tracks for the development of industrial strains and the reduction of the use of chemicals such as cysteine. On the contrary, yeast strains with low NTS activity could help bread-makers to increase dough strength and bread volume. The shelf-life and alimentary properties of “NTS boosted” bread could also reveal further interesting aspects of the Trx system in food, since Trx is not only a regulator of the global redox state of the cell, but also regulates many enzymes that are involved in seed metabolism, such as starch hydrolase limit dextrinase [24].

## Abbreviations

Trx	thioredoxin
NTR	NADPH-dependent thioredoxin reductase
NADPH	Nicotinamide adenine dinucleotide phosphate
GSH	glutathione
NTS	NADPH-dependent NTR and Thioredoxin system
